# Note on Spermatocele with Case1Read at the thirty-second annual meeting of the Medical Association of Central New York, at Syracuse, October 17, 1899.

**Published:** 1899-12

**Authors:** J. Henry Dowd

**Affiliations:** Buffalo, N. Y., Genito-urinary surgeon to Buffalo Hospital Sisters of Charity


### NOTE ON SPERMATOCELE WITH CASE.[1]

By J. HENRY DOWD, M. D., Buffalo, N. Y.,
Genito-urinary surgeon to Buffalo Hospital Sisters of Charity.

THE specimen that I present for your consideration was obtained from a patient giving the following history :

J. H., aged 47, single; occupation, pipe line walker, oilfields, Pennsylvania. Family history negative; personal history and appearance that which is only found in persons whose occupation keeps them continuously out of doors.

About June 15, while walking through a ravine, caught his foot in a twig, falling forward and striking, but lightly, against a fallen tree, which touched the body about its center. At the time he noticed a slight twinge of pain in the right testicle, but nothing that prevented the continuing of his journey. That evening he noticed considerable swelling, but this was painless and gradually disappeared, leaving only a small rounded body, the size of a hickory nut. This swelling persisted, but in no way interfering with his occupation, which consisted in walking about eight or ten miles daily over very rough roads. On July 19th, he came into my ward at the hospital, not for any physical suffering, but for advice and to allay the mental anxiety that the growth might destroy the gland. After a careful examination of testicle, prostate and vesicles a diagnosis of spermatocele was given, with the suggestion that it could be removed and in no way interfere with the sexual function or destroy the gland. Consenting to its removal on July 21st, chloroform was administered and the dartos laid open down to the tunica vaginalis. Here it was found that although the growth lay behind this, it would not be possible to reach it without cutting through the latter. This was done, both layers being divided and the pedicle—if such I may call it—was ligated and cut away from its connection, with the head of the epididymis (vas efferentia). In doing this the knife accidentally cut through the very thin wall, but my house surgeon, Dr. William Gerber, who like many others was very sceptical of the diagnosis, caught the fluid, later examining it and proving the correctness of my opinion.

The two layers of the tunica were sewed with fine gut and the dartos with an uninterrupted suture was closed over all. Healing took place by first intention, the patient being up on the fourth day, and leaving for home three or four days later with only a slight induration of the cord, which rapidly passed away.

Although this growth would appear of trivial importance it should receive most careful consideration for several reasons :

1. It is probably one of the rarest forms of tumor found in the

1. Read at the thirty-second annual meeting of the Medical Association of Central New York, at Syracuse, October 17, 1899.

body and especially of the scrotum. This statement can be substantiated by reference to any authority on genito-urinary surgery.

2. Its liability to rapid growth, attaining at times the size of a cocoanut.

3. Springing as it does from the vas efferentia there is but one result of unobstructed enlargement, atrophy of the testicle. These growths appearing, as they do, in the prime of life, when the sexual function is at its best, atrophy of the testicle can only be followed by that train of symptoms so peculiar and trying to those who treat their possessor.

4. The usually advised treatment, that of tapping, is too often followed by relapse and Volkmann's method, similar to that for hydrocele, necessitates a quite long continuance of dressings, and the like.

These tumors can be carefully dissected from their bed, either from behind or through the tunica vaginalis, as in my case. Much care should be used if our choice is the latter, for healing by first intention is all important in this, preventing scar tissue or testicular adhesions, which are so often followed by glandular (testicle) neuralgia.

288 Franklin Street.

